# Preparative Purification of Anti-Proliferative Diarylheptanoids from *Betula platyphylla* by High-Speed Counter-Current Chromatography

**DOI:** 10.3390/molecules21060700

**Published:** 2016-05-28

**Authors:** Namki Cho, Hyun Woo Kim, Tae Bum Kim, Tanya T. Ransom, John A. Beutler, Sang Hyun Sung

**Affiliations:** 1 College of Pharmacy and Research Institute of Pharmaceutical Science, Seoul National University, Seoul 08826, Korea; cnamki@naver.com (N.C.); kimkami2@snu.ac.kr (H.W.K.); gtato311@snu.ac.kr (T.B.K.); 2 Molecular Targets Laboratory, Center for Cancer Research, National Cancer Institute, Frederick, MD 21702-1202, USA; ransomt@mail.nih.gov (T.T.R.); beutlerj@mail.nih.gov (J.A.B.)

**Keywords:** HSCCC, diarylheptanoid, anti-proliferation

## Abstract

A simple and rapid method using high-speed counter-current chromatography (HSCCC), along with bioassay-guided fractionation based on the anti-proliferative activity against renal and colon cancer cells, has been developed for the preparative separation of aceroside VIII (**1**) and platyphylloside (**2**) from *Betula platyphylla*. A solvent system composed of ethyl acetate/acetonitrile/water (1:0.1:1, *v*/*v*/*v*) was optimized for the separation. The upper phase was used as the stationary phase, and the lower phase was used as the mobile phase. Among these isolated diarylheptanoids, platyphylloside (**2**) showed anti-proliferative activity in the COLO205 and KM12 colon cells and renal cancer cell lines A498, U031, as well as in MG63 and MG 63.3 osteosarcoma cells. In addition, it showed dose dependent inhibitory effects in the NCI 60 cell line assay. These results suggest that the diarylheptanoids isolated from *B. platyphylla* with an efficient HSCCC method could be potential multi-targeted therapeutic agents for cancer.

## 1. Introduction

About 58,000 individuals were diagnosed with renal cancer in 2011, accounting for 3% of the cancers in the United States. [1,2]. Renal cell carcinoma is the most common type of both sporadic and hereditary kidney cancer induced by mutations occurring in the tumor suppressor gene VHL [2]. Colon cancer is also the second-most prevalent cancer among men and women in the United States, and the mortality resulting from this disease is due to metastatic disease in the form of many solid tumors [3]. Colorectal cancer is the development of cancer in the colon or rectum due to the abnormal growth of cells that have the ability to metastaize to other parts of the body [4]. Therefore, it is important to find multi-target agents with anti-proliferative and anti-metastatic potentials in various cancer cells [3,4].

The bark of *B. platyphylla*, a tree widely distributed throughout Korea, is well known as a traditional medicine for arthritis, dermatitis, and nephritis treatment in China, Japan, and Korea [5,6]. Platyphylloside, aceroside VIII, and betulin have been investigated as the three main bioactive constituents and reported to be responsible for the various biological activities of *B. platyphylla* [6,7]. In our previous study, we already reported on the neuroprotective effects of *B. platyphylla* bark extract and its major compounds *in vitro* and *in vivo* [7,8].

Diarylheptanoids, with two aromatic rings joined by a heptane chain, are typical secondary metabolites in the genus *Alnus*, and widely distributed in other genera, such as *Zingiber*, *Curcuma*, *Alpinia*, and *Betula* [9]. These phenolic compounds are derived from two C6-C3 blocks which are connected with one more carbons originating from malonyl CoA [10,11]. It is known that diarylheptanoids possess a wide range of biological inhibitory activities, including antioxidant, anti-inflammatory, antiviral, and anticancer [12,13] properties. In addition, recently, Ryu *et al.* have reported that aceroside VIII from *B. platyphylla* has anticancer activity as a HDAC inhibitor in HT29 cells [14]. In line with previous studies, we evaluated the anti-proliferative activities of two diarylheptanoids against renal cancer cell lines (A498, U031), colon cancer cell lines (COLO205, KM12), and osteosarcoma cell lines (MG63, MG63.3). In addition, the compounds were tested in the NCI 60-cell screen in order to assess their cytotoxic profile.

**Figure 1:**
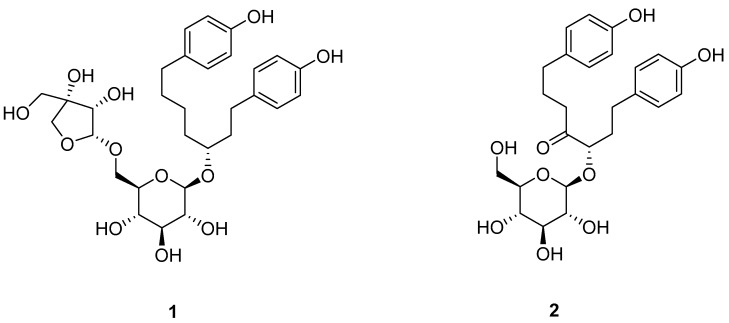
Chemical structure of two diarylheptanoids from *B. platyphylla* bark: aceroside VIII (**1**), and platyphylloside (**2**).

Conventional isolation techniques for these diarylheptanoids require multiple chromatographic steps, which are time consuming and result in sample loss due to irreversible adsorption [15]. However, high-speed counter-current chromatography (HSCCC), a liquid–liquid partition chromatographic technique, is capable of isolating multiple components from plant extracts without irreversible adsorption, since there is no solid phase involved [16,17]. Due to the advantages of HSCCC, such as the lack of irreversible adsorption, total recovery of the injected sample, minimized tailing, and low solvent consumption, it has been widely used in the preparative separation and purification of various compounds from natural products [18,19]. Effective application of HTS to bioactive extracts and the development of hits from screening campaigns into leads for drug development are clearly important to access anticancer lead compounds in secondary metabolites of natural products. [20]. To the best of our knowledge, there are few reports on the preparative separation of diarylheptanoids from plant extracts using HSCCC to date [21,22]. The present paper describes the successful preparative separation and purification of the two diarylheptanoids aceroside VIII (**1**) and platyphylloside (**2**) (Figure 1) from a crude sample of *B. platyphylla* using HSCCC in stepwise elution mode.

## 2. Results and Discussion

### 2.1. Effect of B. platyphylla Bark Extract and Fractions on Renal and Colon Cancer Cells

**Figure 2:**
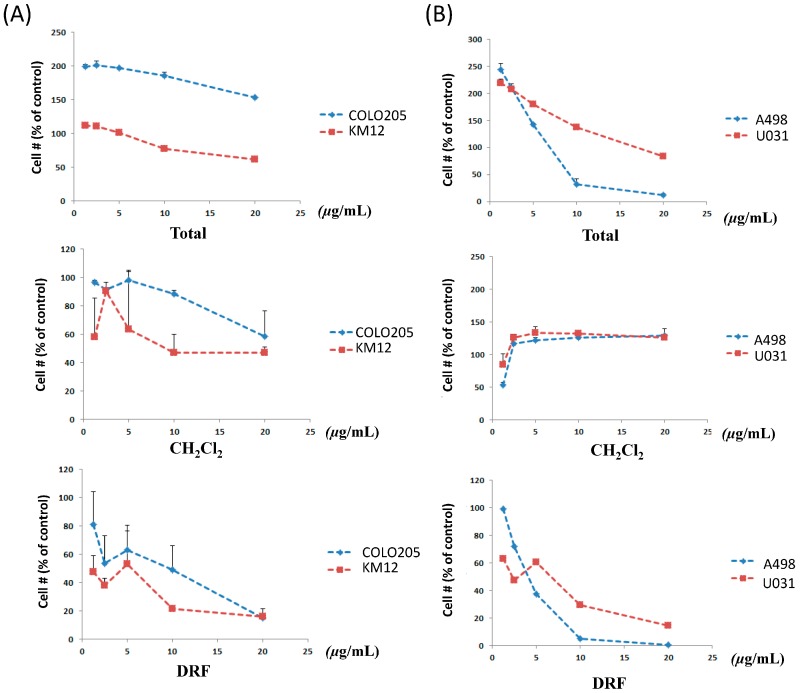
Cytotoxicity of total extract, CH_2_Cl_2_ fraction, and DRF (diarylheptanoid-rich fractions) in colon tumor cells COLO205, KM12 (**A**) renal tumor cells A498, U031; (**B**) cell number was calculated as (A_405_ of sample treated − A_405_ of no cell control)/(A_405_ of DMSO control − A_405_ of no cell control) × 100%.

We observed that a total extract of *B. platyphylla* bark showed an inhibitory effect on renal and colon cancer cells in a dose-dependent manner. In order to isolate and identify the bioactive compounds, we carried out fractionation of the methanol extract of *B. platyphylla* bark. The diarylheptanoid-rich fraction (DRF) exhibited anti-proliferative activity (IC_50_ (μg/mL) values for COLO 205 (9.6) and KM12 (5.4), A498 (6.7), and U031 (3.8)) (Figure 2) where as the CH_2_Cl_2_ fraction has no effects, even though it could not inhibit cell proliferation at high concentration. Therefore, we focused on obtaining the major bioactive diarylheptanoids using only HSCCC separation for a rapid, large-scale isolation.

### 2.2. Solvent System Selection

Selection of a suitable solvent system is the most important step in successful separation by HSCCC. According to rules previously outlined by Ito, the ideal partition coefficient *K*_D_ values of the target compound should be between 0.5 and 2 [19,22]. Furthermore, the separation factor (α), which is the ratio of the two *K*_D_ values, should be greater than 1.5 for resolution between the target compounds [20,21].

In this study, several conditions were tested for suitability, and two-phase solvent systems, ethylacetate/methanol/H_2_O and ethylacetate/acetonitrile/H_2_O, showed good values for the HSCCC separation. As shown in Table 1, the first solvent system ethyl acetate/butanol/water (1:0.1:1) had a large *K* value for the target compounds, which could produce excessive band broadening due to a longer elution time. Since the hydrodynamic equilibrium of the second solvent system, which is ethylacetate/methanol/water (1:0.1:1), was not reached, the solvent condition comprised of ethylacetate/acetonitrile/H_2_O (1:0.1:1, *v*/*v*/*v*) was selected to purify compound **1** (*K* = 1.08) and compound **2** (*K* = 1.67).

### 2.3. HSCCC Separation

**Figure 3:**
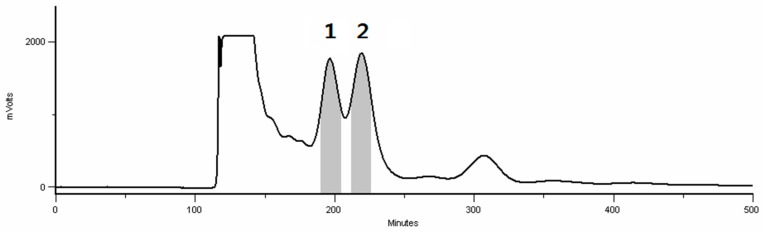
Chromatograms of HSCCC obtained from the diarylheptanoid-rich fractions of *B. platyphylla* using the two-phase solvent system ethylacetate–acetonitrile–water 1:0.1:1 (*v*/*v*/*v*). The stationary phase was the upper phase, the mobile phase the lower phase, and the flow rate was 1.5 mL/min. The revolution speed was 1000 rpm. The sample size was 100 mg, and the separation temperature 25 °C.

**Figure 4:**
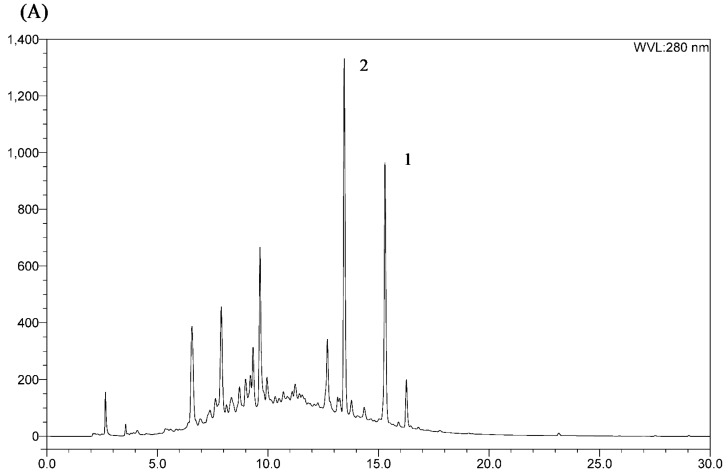

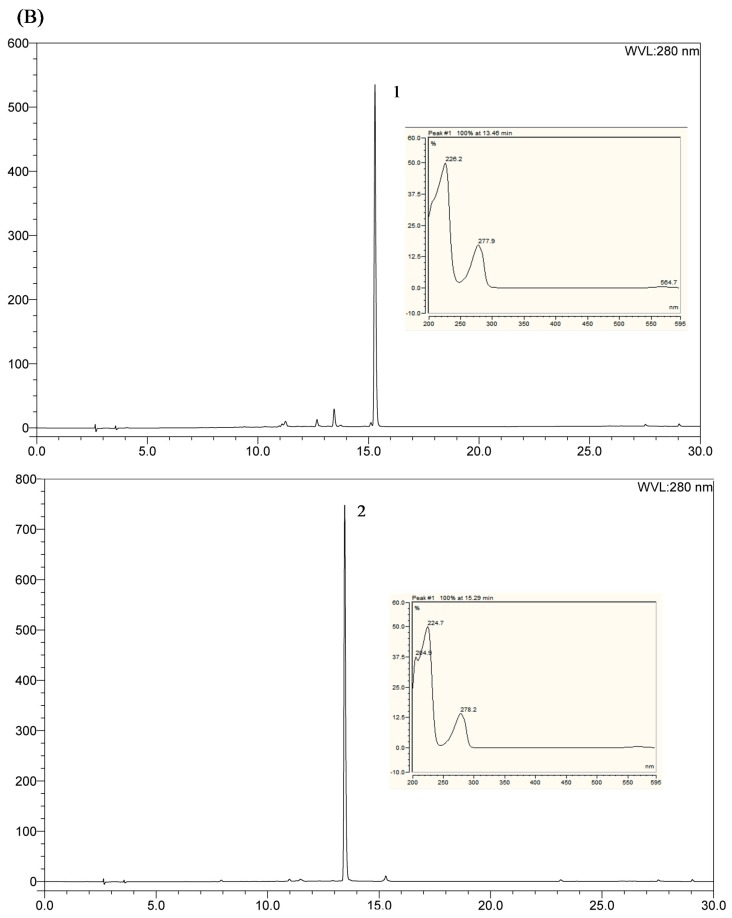
Chromatograms of HPLC of the DRF (**A**) and collected compounds (**B**) from HSCCC. Experimental condition of HPLC: a Capcell Pak C-18 column (150 mm × 4.6 mm, I.D., 5 μm, Shiseido Co., Ltd., Tokyo, Japan.), with the following gradient system was used: water with 0.1% formic acid (*v*/*v*) (**A**) and acetonitrile (**B**); gradient system: 0–25 min, 10%–50% B; 25–30 min, 50%–100% B.

After the removal of the CH_2_Cl_2_ fraction, the DRF (100 mg) was subjected to HSCCC under our optimized conditions. From the HSCCC chromatogram shown in Figure 3, two fractions were obtained, and two major diarylheptanoids were isolated: aceroside VIII (Compound **1**, 15 mg) and platyphylloside (Compound **2**, 10 mg). The crude sample and peak fractions obtained by HSCCC were analyzed by analytical HPLC, with the chromatograms shown in Figure 4. The purity of the compound measured by the HPLC chromatogram was 95.2% and 96.0%, respectively. The results clearly show that HSCCC provides a highly efficient preparative separation of diarylheptanoids from *B. platyphylla* bark.

### 2.4. Comparison with HSCCC Separation and Traditional Isolation Techniques

Comparing to the previous traditional isolation techniques, our methodology using HSCCC was more efficient in many ways [23]. First, we performed only two steps, extraction and HSCCC separation, while previous reported isolation was comprised of many steps, including the fractionation with CHCl_3_, *n*-BuOH and H_2_O, separation with Diaion HP-20, column chromatography separation with MPLC. and recrystallization. Furthermore, the yield of compound **1** and **2** from HSCCC separation was also better than that of traditional isolation technique. In HSCCC separation, compound **1** (15 mg) and **2** (10 mg) were isolated from 200 mg of methanolic extract, showing yields of 7.5% and 5% each, in contrast of low yield under 3.0% using the traditional method. These results demonstrated that HSCCC separation was a time-saving and efficient methodology for isolation of two major diarylheptanoids from *B. platyphylla*.

### 2.5. Anti-Proliferative Effects

**Figure 5:**
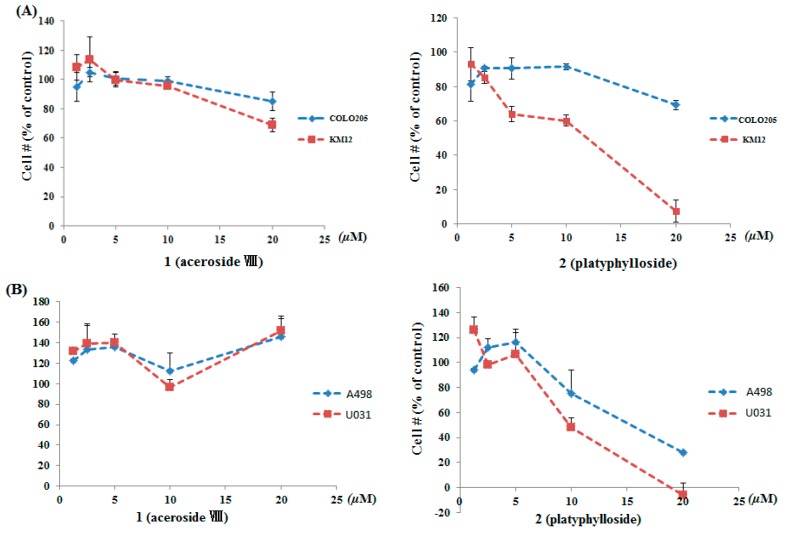

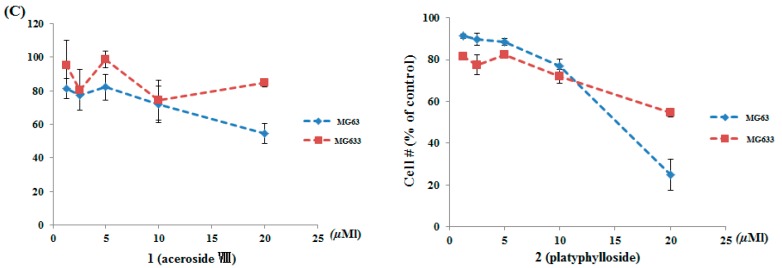
Cytotoxicity of compounds aceroside VIII (**1**) and platyphylloside (**2**) in colon tumor cells COLO205, KM12 (**A**) renal tumor cells A498, U031 (**B**) and osteosarcoma cells MG63 and MG63.3 (**C**). Cell proliferative activity was determined by XTT assay, and data are the mean ± SD of the percentage of cell proliferation from three independent experiments.

We investigated the inhibitory activity of two-day drug exposure against colon and renal cancer, and osteosarcoma metastatic potential assays, using high-throughput screening (HTS) assays developed at the NCI (National Cancer Institute) [5,24,25]. Previously Ju *et al.* reported the induced apoptotic cell death in human promyelocytic leukemia (HL-60) cells, a cancer cell line with treatment of the total methanol extract of *B. platyphylla* at concentrations from 4.0 to 500.0 μg/mL [26]. Here, we narrowed the total extract down to an active fraction by HSCCC, showed to be more anti-proliferative of DRF at lower concentrations, from 1.25 to 20.0 μg/mL. By using bioassay-guided isolation with HSCCC based on the anti-proliferative activity against renal and colon cancer cells, we obtained two major diarylheptanoids, aceroside VIII (**1**) and platyphylloside (**2**). Our results show that platyphylloside (**2**) inhibited cell growth of KM12 colon cancer cells in a dose-dependent manner (Figure 5A). At a concentration ranging from 1.25 μM to 20 μM, platyphylloside exhibited a significant antip-roliferative activity (IC_50_ 11.8 μM), which was more potent than that of aceroside VIII (IC_50_ > 20.0 μM). Platyphylloside potently inhibited cell growth by 93% at 20 μM. It also showed potent anti-proliferative activity in A498 and U031 in a dose-dependent manner (Figure 5B). Platyphylloside, with an IC_50_ value (17.5 and 16.6 μM), was superior in renal cell lines to aceroside VIII (IC_50_ > 20.0 μM) despite the structural similarity. The slight structural differences, such as the presence of one more sugar moiety at C-6 and the absence of a ketone group in the aliphatic chain seemed to affect the inhibitory activity. To investigate the anti-metastatic potentials of compounds aceroside VIII (**1**) and platyphylloside (**2**) on the growth of cancer cell lines, we further evaluated the cytotoxicity of high (MG63) and low metastatic (MG63.3) potential osteosarcoma cell lines with the XTT assay (Figure 5C). Platyphylloside (**2**) showed cell growth inhibition with an IC_50_ value of 15.1 μM in MG63, but did not show more growth inhibition to the more highly metastatin MG63.3 cell line.

**Figure 6:**
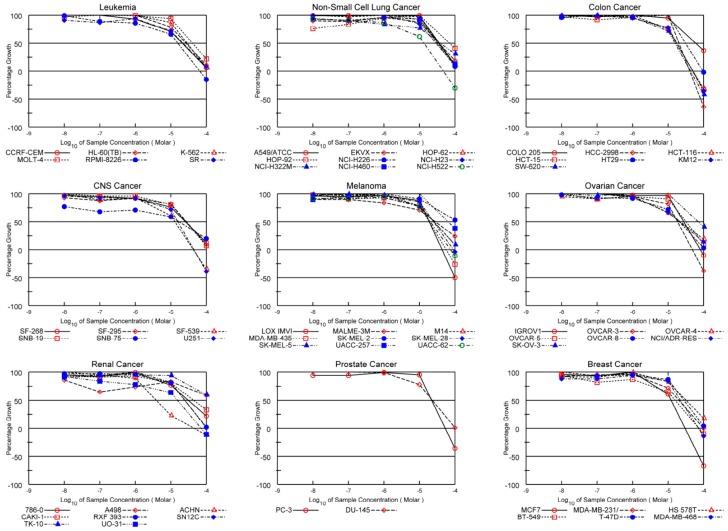
The anti-tumor effect of platyphylloside (**2**) in the NCI-60 cancer cell lines in a 5-dose dependent manner. The cytotoxic effect of platyphylloside (**2**) in the NCI-60 tumor cell panel shows a preference for renal cancer cell lines.

To further assess the biological effect of these diarylheptanoids on the viability of tumor cells, we used the NCI-60 screen, a platform containing 60 different cancer cell lines (Figures S1–S5) [25,27]. We used adriamycin, which is well known as cytotoxic, as positive control. In line with the results of the two-day assay, platyphylloside (**2**) showed selectivity towards human colon cancer lines, such as HCT-116, HCT-15, and HT-29 with 72 mean percent in NCI 60 cell line one-dose test. (Figures S1 and S2 in Supplementary Materials, Table 2). It also showed selectivity towards ACHN and UO-31 renal cancer cell lines in a five dose experiment (Figure 6). This tabular data of platyphylloside could be comparable to that of adriamycin. It might be worthy, as diarylheptanoids are found in several plants consumed by people as food and medicines, while adriamycin probably causes serious cardiac toxicity.

## 3. Experimental Section

### 3.1. Materials and Reagents

The dried bark of *B. platyphylla* was collected at the afforested land of SK E & C (Seoul, Korea) and ground into powder. A voucher specimen was deposited at the College of Pharmacy, Seoul National University, Korea. All organic solvents and water used for the sample preparation and HSCCC were analytical grade and purchased from the Daejung Chemical & Metals CO., Ltd. (Gyeonggi-Do, Korea). The bark of *B. platyphylla* (32.0 g) was extracted three times with 80% methanol in an ultrasonic apparatus at room temperature. After removal of the solvent in vacuo, the 80% methanolic extract (3.0 g) was suspended in H_2_O and partitioned into CH_2_Cl_2_ generating betulin- (1.4 g) and a diarylheptanoid-rich fraction (DRF) (1.5 g).

### 3.2. Apparatus

The preparative HSCCC system used in this study consisted of a TBE-300A (Shanghai Tauto Viotech, C., Ltd., Shanghai, China) equipped with PTFE tubing (I.D. = 3.0 mm; total volume = 300 mL), a chromatographic pump (L-6200, Hitachi, Tokyo, Japan), a UV detector (UV/VIS-151, Gilson Inc., Middleton, WI, USA), and the Unipoint^®^ data system software (5.11, Gilson Inc., Middleton, WI, USA). The HSCCC system was kept at an internal column temperature of 25 °C with a circulatory temperature regulator (RW-0525G, Jeio Tech., Seoul, Korea). Fractions were collected with a fraction collector FC-204 (Gilson). The analytical HPLC-UV system consisted of a Dionex HPLC system with a P680 HPLC pump, a Dionex ASI-100 autosampler and a Dionex UVD340U UV detector (Thermo Fisher Scientific Inc., Waltham, MA, USA). The column used in this work was a Capcell Pak C-18 column (150 mm × 4.6 mm, I.D., 5 μM, Shiseido Co., Ltd., Tokyo, Japan). ESI/MS spectra (Finnigan LCQ advantage MS, Thermo®, San Jose, CA, USA) and ^1^H- and ^13^C-NMR spectra (JEOL ECA-500, JEOL Ltd., Tokyo, Japan) were obtained by analysts at the Research Institute of Pharmaceutical Sciences, Seoul National University.

### 3.3. HPLC Analysis

The crude extract and each peak fraction separated by HSCCC were analyzed by HPLC. The solvent system was composed of 0.1% formic acid in water (A) and acetonitrile (B). The gradient condition was as follows: 0–25 min, 10%–50% B; 25–30 min, 50%–100% B. The column oven temperature was set at 25 °C. The flow rate was 1.0 mL/min, and 10 μL aliquots were injected into the column. The chromatogram was measured at 280 nm. The HPLC-UV spectroscopic data were obtained on a Dionex HPLC system with the Chromeleon^®^ chromatographic software (7.0, Dionex Corp., Germering, Germany).

### 3.4. Determination of the Partition Coefficient Values (K-Value)

Approximately 5 mg of extract were weighed in a 20 mL test vial, and each phase of the pre-equilibrated two phase solvent system was added. The test vial was shaken violently for several minutes to achieve equilibrium. The upper and lower phases were separated and evaporated under N_2_ gas. The dried residues of each phase were dissolved in an acetonitrile/water mixture (1:1, *v*/*v*) of 1 mL, and analyzed by HPLC-UV. The *K*-value was obtained with the ratio of the peak area between the upper and lower phase.

### 3.5. Preparation of the Two-Phase Solvent System and Sample Solution

After determining the *K*_D_ value by HPLC-UV, the two-phase solvent system ethyl-acetate/acetonitrile/H_2_O (1:0.1:1, *v*/*v*/*v*) was selected. The two-phase solvent system was equilibrated in a separation funnel by repeated vigorous shaking at room temperature and only separated shortly before use.

### 3.6. HSCCC Separation Procedure

The mobile phase was selected as the aqueous lower phase. The multilayer coil column was filled first with the organic upper phase as the stationary phase. Then, the mobile upper phase was pumped into the column at a flow rate of 1.5 mL/min, while the preparative HSCCC apparatus was rotated at 1000 rpm. After hydrodynamic equilibrium was established, BRF was injected into the HSCCC. The monitoring of HSCCC peak fraction was done by combining the effluent line of the HSCCC to the UV detector at 280 nm. The eluent from the UV detector was collected by a fraction collector every 5 min per test tube.

### 3.7. Identification of Isolated Peak Fraction

HSCCC peak fractions were identified by comparing their ^1^H-, ^13^C-NMR, and ESI-Q-TOF MS spectroscopic data with the literature data. ^1^H- and ^13^C-NMR spectroscopic data were obtained on an AVANCE 400 WB spectrometer at 400 MHz and 100 MHz, respectively. The ESI-Q-ToF MS spectroscopic data were obtained on a Waters Xevo G2 QTOF mass spectrometer (Waters MS Technologies, Manchester, UK).

*Compound* **1***.* Pale yellowish amorphous powder, ESI-Q-TOF (*m*/*z*): 593.2623 [M − H]^−^, C_30_H_42_O_12_^1^H-NMR (500 MHz, CD_3_OD); δ 6.98 (2H, d, *J* = 8.4 Hz, H-2′,2′′,6′,6′′), 6.68 (2H, d, *J* = 8.4 Hz, H-3′,3′′,5′,5′′), 3.27–3.38 (1H, m, H-3), 2.58 (2H, m, H-1), 2.47 (2H, t, *J* = 7.5 Hz, H-7), 1.75 (2H, m, H-2), 1.59 (2H, m, H-4), 1.52 (2H, m, H-6), 1.35 (2H, m, H-5). Glucose; 4.26 (1H, d, *J* = 7.5 Hz, H-1), 4.00 (1H, br d, *J* = 11.6 Hz, H-6b), 3.60–3.68 (1H, m, H-6a), 3.60–3.68 (1H, m, H-3), 3.27–3.38 (2H, m, H-4, H-5), 3.19 (1H, m, H-2). Apiose; 5.01 (1H, d, *J* = 2.2 Hz, H-1), 3.92 (1H, d, *J* = 9.4 Hz, H-2), 3.91 (1H, m, H-4), 3.76 (1H, d, *J* = 9.6 Hz, H-4), 3.55 (2H, d, *J* = 11.5 Hz, H-5). ^13^C-NMR (125 MHz, CD_3_OD); δ 156.8 (C-4′,4′′), 135.6 (C-1′,1′′), 131.1 (C-2′,2′′,6′,6′′), 116.9 (C-3′,3′′,5′,5′′), 78.8 (C-3), 38.9 (C-2), 36.6 (C-7), 35.7 (C-4), 33.8 (C-6), 32.4 (C-1), 26.4 (C-5). Glucose; 104.2 (C-1), 80.7 (C-3), 77.3 (C-5), 76.0 (C-2), 72.3 (C-4), 69.1 (C-6). Apiose; 111.6 (C-1), 81.3 (C-3), 78.8 (C-2), 75.8 (C-4), 66.5 (C-5). By comparison with the literature data [28], compound **1** was identified as aceroside VIII.

*Compound* **2**. Pale brownish amorphous powder, ESI-Q-TOF (*m*/*z*): 475.1961 [M − H]^−^, C_25_H_32_O_9_^1^H-NMR (500 MHz, CD_3_OD); δ 6.99 (2H, d, *J* = 8.0 Hz, H-2′,2′′,6′,6′′), 6.67 (2H, d, *J* = 7.8 Hz, H-3′,3′′,5′,5′′), 4.16 (1H, q, *J* = 5.6 Hz, H-5), 2.79 (1H, dd, *J* = 16.6, 6.8 Hz, H-4b), 2.73 (4 H, s, H-1,2), 2.52–2.63 (1H, m, H-4a), 2.52–2.63 (2H, m, H-7), 1.66–1.89 (2H, m, H-6), Glucose; 4.3 (1H, d, *J* = 7.7 Hz, H-1), 3.86 (1H, dd, *J* = 11.8, 2.2 Hz, H-6b), 3.69 (1H, dd, *J* = 11.8, 5.3 Hz, H-6a), 3.30–3.38 (1H, m, H-3), 3.30–3.38 (2H, m, H-4, H-5), 3.15 (1H, m, H-2). ^13^C NMR (125 MHz, CD_3_OD); δ 212.7 (C-3), 157.1 (C-4′,4′′), 135.1 (C-1′,1′′), 131.1 (C-2′,2′′,6′,6′′), 116.8 (C-3′,3′′,5′,5′′), 77.0 (C-5), 49.5 (C-4), 47.2 (C-2), 39.3 (C-6), 32.2 (C-7), 30.6 (C-1). Glucose; 104.2 (C-1), 78.8 (C-3), 78.6 (C-5), 76.0 (C-2), 72.4 (C-4), 63.5 (C-6). By comparison with the literature data [29], compound **2** was identified as platyphylloside.

### 3.8. Cytotoxicity Assay for Colon, Renal, and Osteosarcoma Cancer Cells

The assay used in this study was an *in vitro* antitumor assay using the XTT endpoint developed by the MTL (Molecular Targets Laboratory) in the Assay Development and Screening section of the NCI. The colon cancer cell lines used were COLO205 and KM12, and the osteosarcoma cancer cell lines were MG63 (low metastatic potential) and MG 63.3 (high metastatic potential). Cells were maintained and passed weekly in RPMI or DMEM medium with phenol red (Gibco, Carlsbad, CA, USA) and supplemented with 2 mM l-glutamine (Quality Biologicals, Inc., Gaithersburg, MD, USA) and 10% fetal bovine serum (Hyclone, Logan, UT, USA). All work was done under sterile conditions using a laminar air-flow hood with external venting. Cells were placed in a humidified incubator with an atmosphere of 5% CO_2_ and 95% air at a temperature of 37 °C. Harvested cells were counted using a Cellometer Auto T4 cell counter (Nexcelom Bioscience LLC, Lawrence, MA, USA) and plated in 384-well flat-bottom polystyrene microtiter plates (Nunc, Nunc A/S, Roskilde, Denmark) at a density of 5000 cells/well for A498, U031, COLO205, and KM12 and 2500 cells/well for MG 63 and MG 63.3. The cells were incubated in a 5% CO_2_ and 95% air atmosphere in a 37 °C incubator for 24 h. After incubation, test samples were added to the plates using a Biomek FX robotic liquid handling workstation (Beckman/Coulter, Fullerton, CA, USA). The robot performed eight two-fold serial dilutions of the sample and then transferred the sample from the source plate to the assay plate. The plates used were Costar 384-well round-bottom plates (Corning Inc., Corning, NY, USA). Cells were further incubated with the samples for 48 h, at which time the XTT reagent was added. A NCI (US National Cancer Institute, Frederick, MD, USA) 60 human tumor cell line anticancer drug screen was performed as previously reported [26]. The positive control standard was adriamycin (NSC#123127).

## 4. Conclusions

Diarylheptanoids, composed of two aromatic rings (aryl groups) joined by a seven carbon chain (heptane), are naturally occurring and abundant in various bushes and trees. The results of this study suggest that HSCCC is an efficient method for the separation and purification of diarylheptanoids from *B. platyphylla* bark using a two-phase solvent system. In addition, we showed, for the first time, the anti-proliferative potential of two major diarylheptanoids from *B. platyphylla* in colon, renal, and osteosarcoma cancer cell lines, as well as their anti-proliferative potential against 60 cancer cell lines. Further studies are needed to investigate the mammalian metabolites of these diarylheptanoids and, if proven safe for clinical use, the industrial-scale extractive isolation of diarylheptanoids from *B. platyphylla* bark could provide a large supply of raw materials for the production of anti-cancer agents.

## Figures and Tables

**Table 1:** *K* values and separation factors of compounds aceroside VIII (**1**) and platyphylloside (**2**).

Solvent System	*K*-Value		Separation
	1	2	Factor
Ethylacetate:Butanol:H_2_O (1:0.1:1, *v*/*v*/*v*)	2.98	3.71	1.25
Ethylacetate:Methanol:H_2_O (1:0.1:1, *v*/*v*/*v*)	0.96	1.52	1.58
Ethylacetate:Acetonitrile:H_2_O (1:0.1:1, *v*/*v*/*v*)	1.08	1.67	1.54

**Table 2:** Cytotoxicity of compounds in the NCI 60-cell screen.

Compound	Mean Percent Inhibition at 10^−5^ M	Percent Range at 10^−5^ M	GI_50_ against COLO205 (μM)	GI_50_ against KM12 (μM)	GI_50_ against A498 (μM)	GI_50_ against U031 (μM)
Total extract	NT ^a^	NT	>20	>20	9	>20
MC	NT	NT	>20	9.1	>20	>20
DRF	NT	NT	9.6	5.4	3.8	6.7
aceroside (**1**)	3	41	>20	>20	>20	>20
platyphylloside (**2**)	72	166	>20	11.8	17.5	16.6
Adriamycin ^b^	40.0 ± 1.3 ^b^ (*n* = 16)	105.5 ± 5.6 ^b^	NT	NT	NT	NT

^a^ NT: Not tested; ^b^ one dose test at 2.5 × 10^−7^ M.

## Supplementary Materials

Supplementary materials can be accessed at: http://www.mdpi.com/1420-3049/21/6/700/s1.
