# Supplementary material for: Preparative Purification of Anti-Proliferative Diarylheptanoids from Betula platyphylla by High-Speed Counter-Current Chromatography
Source: Molecules. 2016 May 28;21(6):700. doi: 10.3390/molecules21060700 (PMC6273792; doi:10.3390/molecules21060700)
Supplement: Supplementary file 1 [file molecules-21-00700-s001.pdf]

Supplementary Materials: Preparative Purification of Antiproliferative Diarylheptanoids from *Betula platyphylla* by High Speed Counter-Current Chromatography

Namki Cho, Hyun Woo Kim, Tae Bum Kim, Tanya T. Ransom, John A. Beutler and Sang Hyun Sung

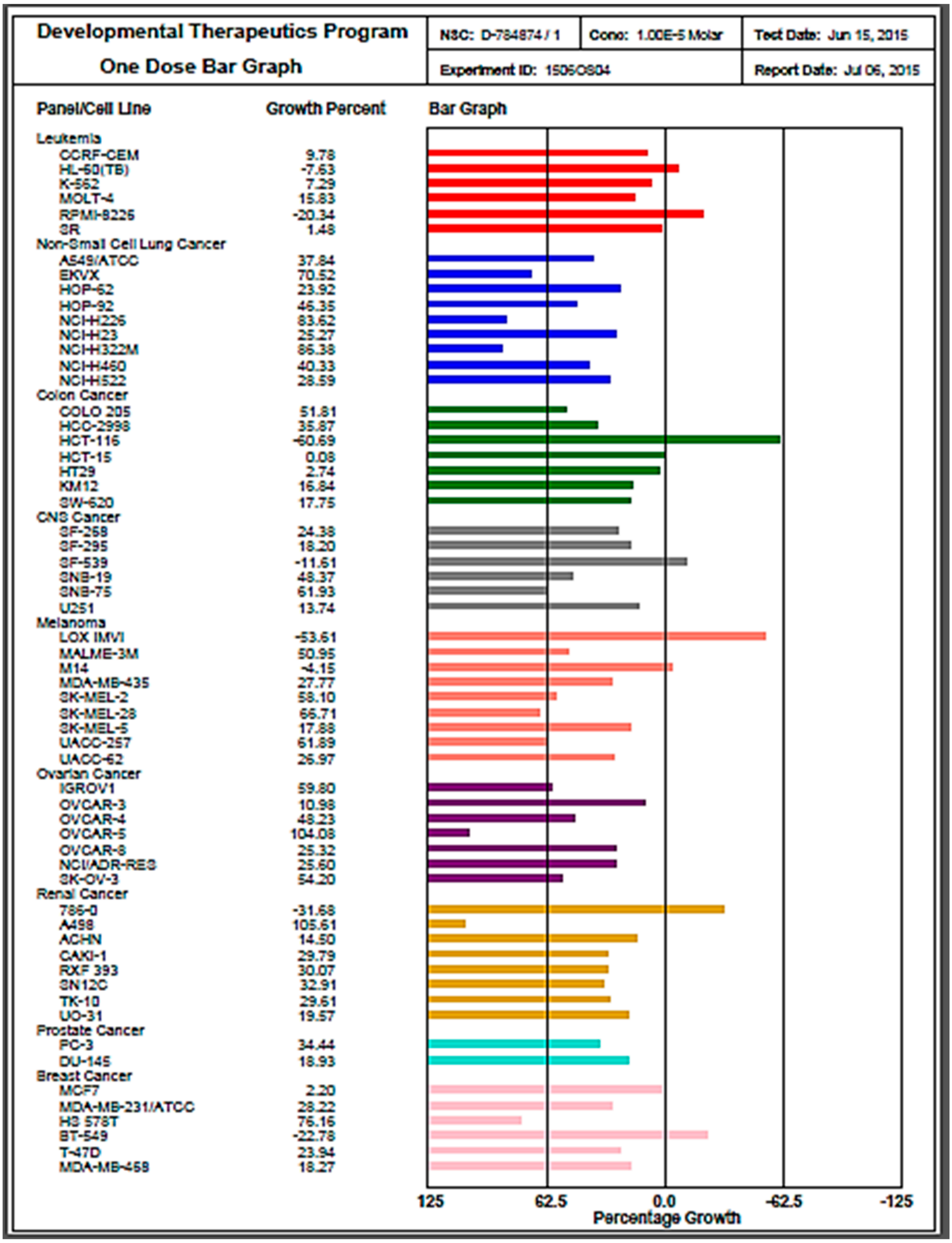

Figure S1. NCI 60 cell single dose test of Platyphylloside (**2**).

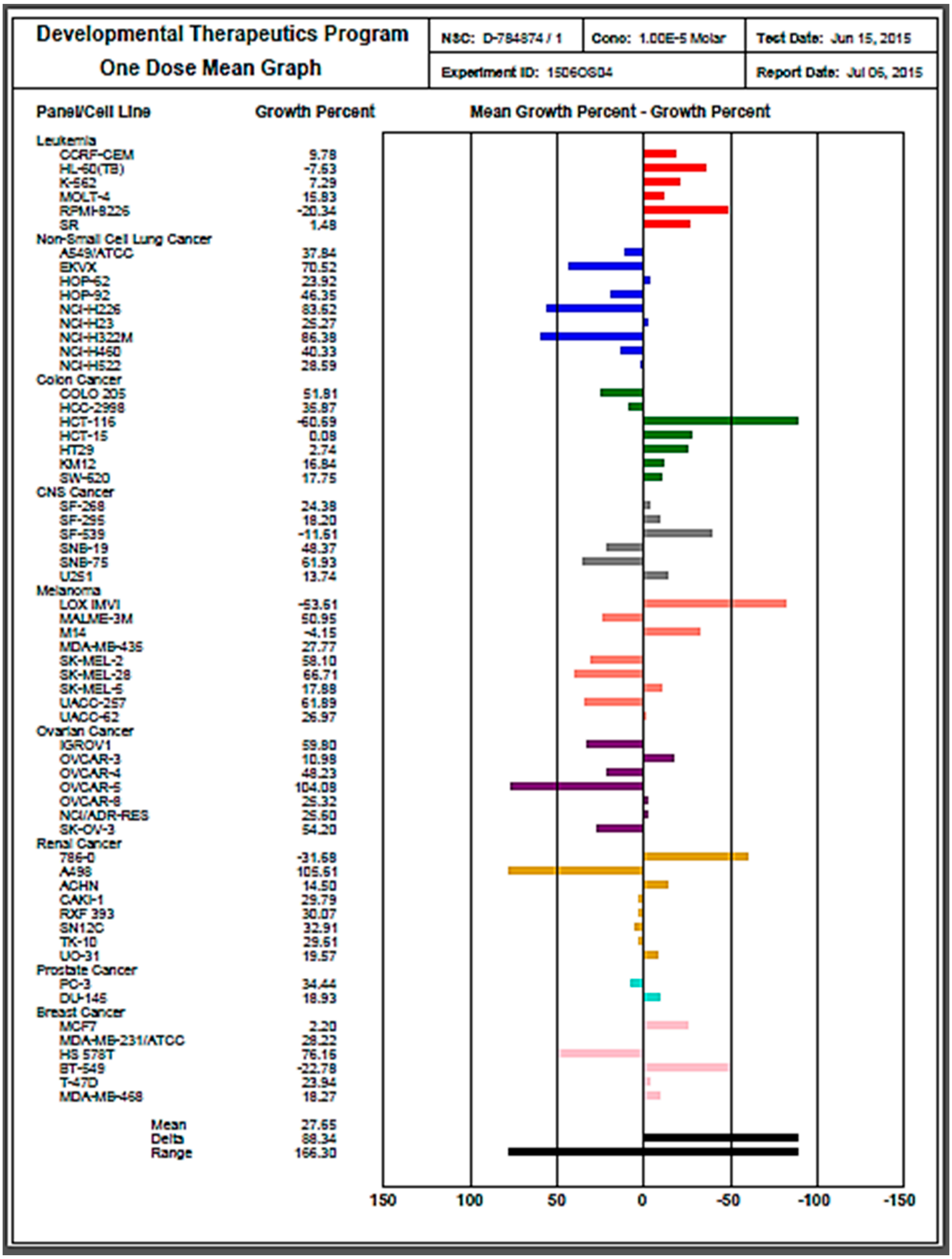

Figure S2. Platyphylloside (**2**) NCI-60 mean bar graphs in single dose test.

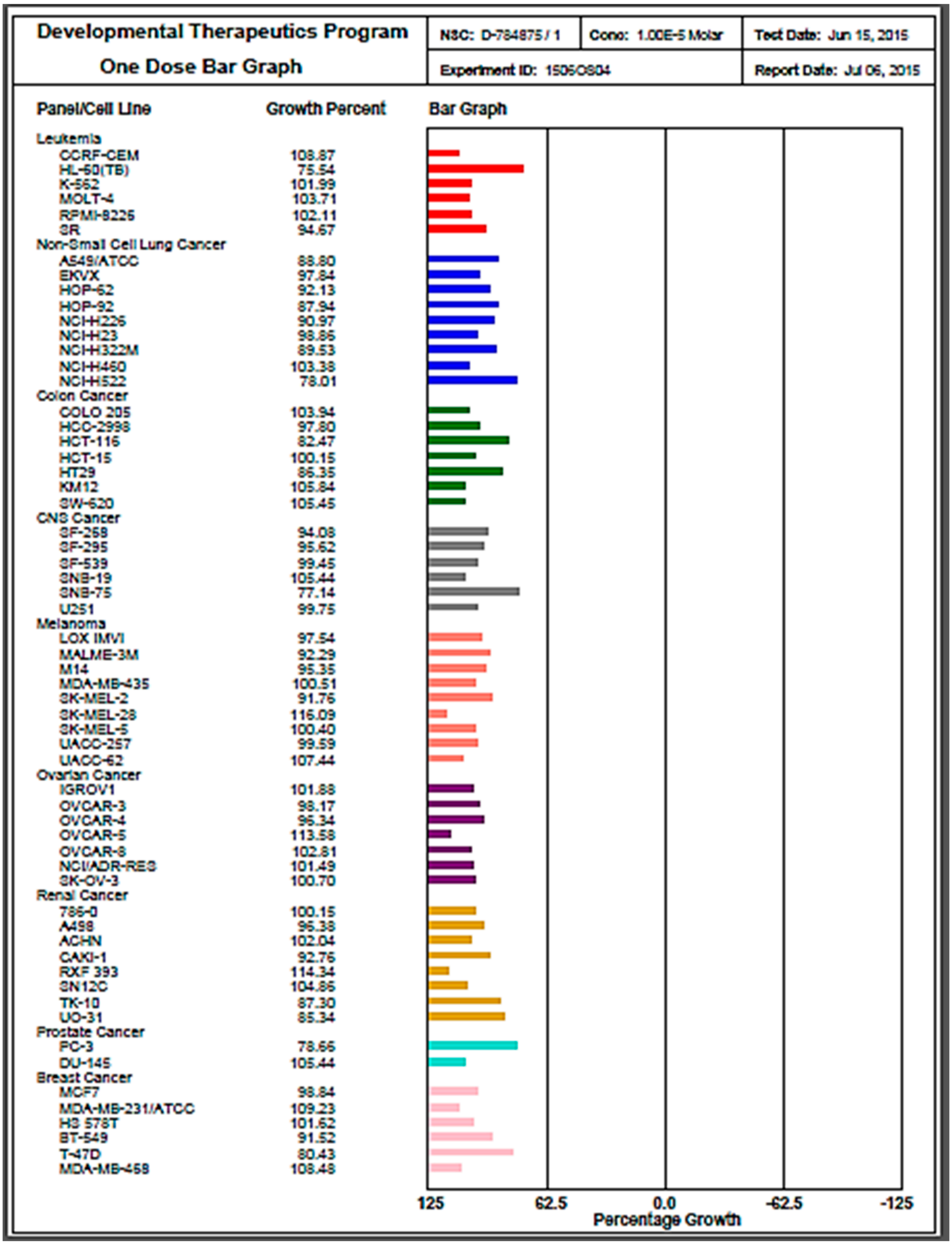

Figure S3. NCI 60 cell single dose test of aceroside VIII (**1**).

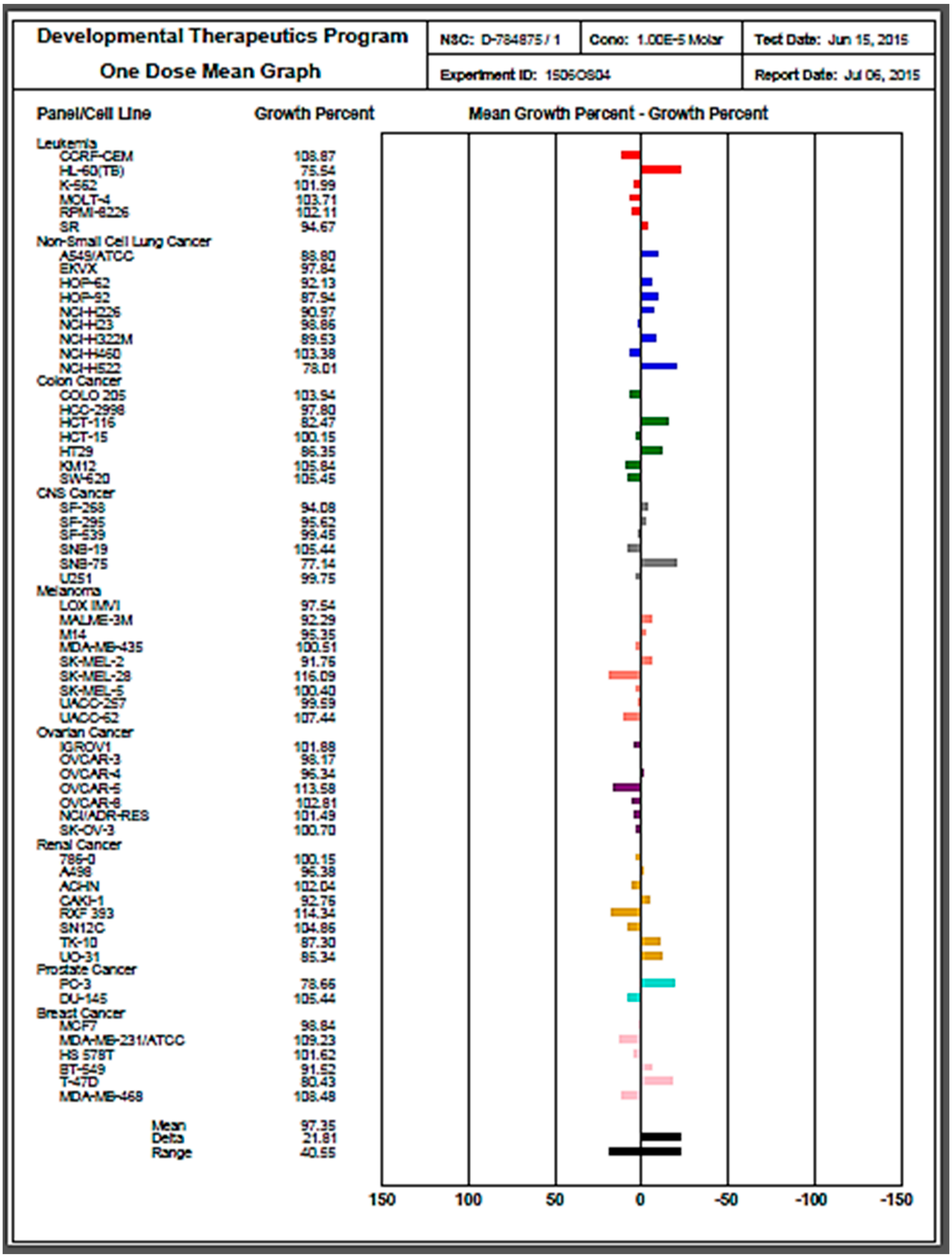

Figure S4. Aceroside VIII (**2**) NCI-60 mean bar graphs.

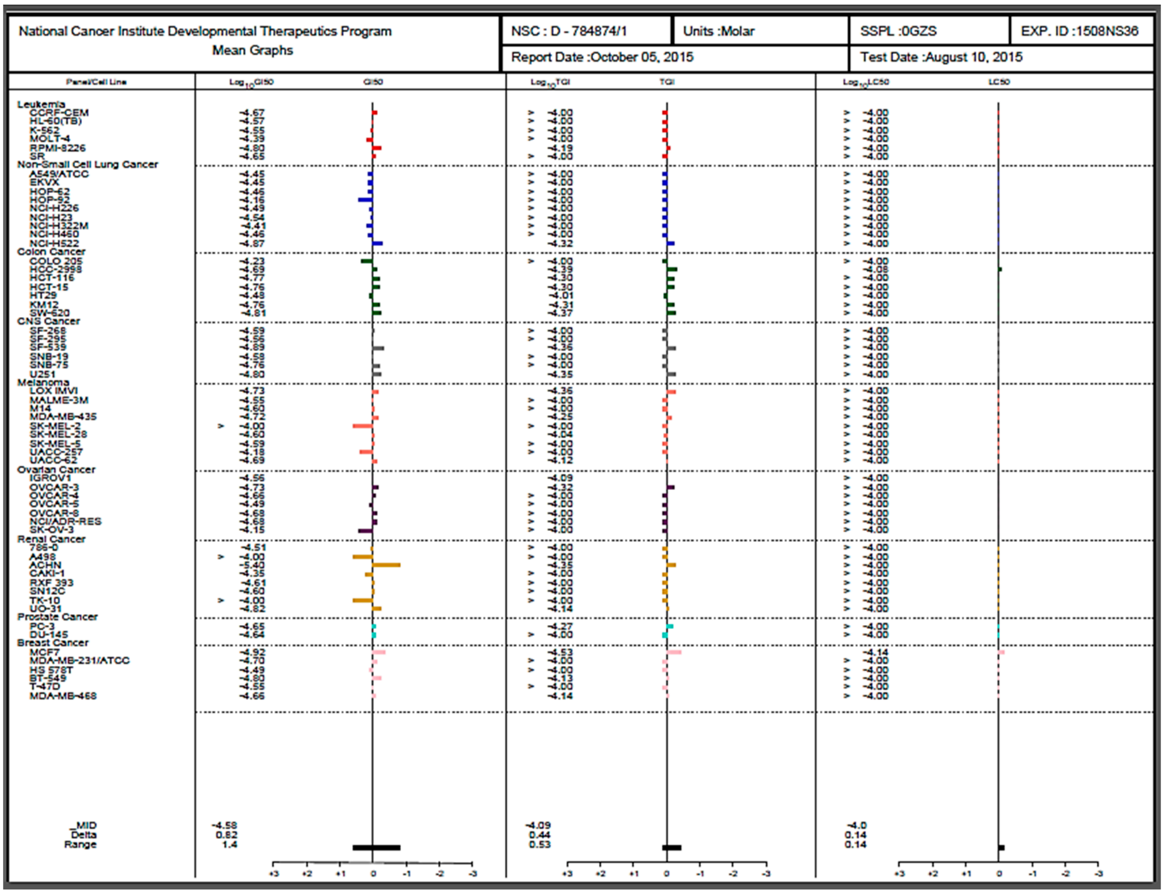

Figure S5. Platyphylloside (**2**) NCI-60 mean bar graphs in five dose-dependent test.
